# Downregulation of Nuclear-Encoded Genes of Oxidative Metabolism in Dialyzed Chronic Kidney Disease Patients

**DOI:** 10.1371/journal.pone.0077847

**Published:** 2013-10-28

**Authors:** Gianluigi Zaza, Simona Granata, Valentina Masola, Carlo Rugiu, Francesco Fantin, Loreto Gesualdo, Francesco Paolo Schena, Antonio Lupo

**Affiliations:** 1 Renal Unit, Department of Medicine, University-Hospital of Verona, Verona, Italy; 2 Section of Geriatrics, Department of Medicine, University-Hospital of Verona, Verona, Italy; 3 Renal, Dialysis and Transplant Unit, Department of Emergency and Transplantation, University of Bari, Bari, Italy; National Cancer Institute, United States of America

## Abstract

**Background:**

Mitochondria, essential eukaryotic cells organelles defined as the “powerhouse of the cell” because of their ability to produce the vast majority of energy necessary for cellular metabolism, may have a primary role in the oxidative stress-related intracellular machinery associated to chronic kidney disease (CKD).

**Methods:**

To better assess this research assumption, we decided to study the key factors regulating mitochondrial oxidative metabolism in CKD patients in peritoneal dialysis (PD, n = 15) using several bio-molecular methodologies.

**Results:**

RT-PCR experiments demonstrate that the expression level of peroxisome proliferator-activated receptor gamma coactivator 1 alpha (*PGC-1α*) and nuclear respiratory factor-1 (*NRF-1)*, two genes primarily involved in mitochondrial biogenesis and functions, were significantly hypo-expressed in peripheral blood mononuclear cells of PD patients compared to healthy subjects (HS, n = 15). Additionally, mRNA levels of several PGC1-α downstream target genes (*TFAM, COX6C,COX7C, UQCRH* and *MCAD*) were profoundly down-regulated in PD cells. TFAM protein analysis confirmed gene-expression results. High plasmatic concentration of Malondialdehyde found in PD patients, confirmed the contribution of the oxidative stress to these biological effects. Finally, Nuclear factor erythroid-derived 2-like 2 (*NRF2 or NFE2L2)*, a transcription factor for numerous antioxidant/detoxifying enzymes and one of its target genes, superoxide dismutase-2 mitochondrial (SOD2) were up-regulated in PD compared to HS.

**Conclusions:**

Our results revealed, for the first time, that CKD-PD patients’ PBMC, through a complex intracellular biochemical machinery, are able to modulate their mitochondrial functions probably in the attempt to reduce oxidative metabolic damage and to turn on a valuable defense cellular strategy against oxidative stress.

## Introduction

Chronic kidney disease (CKD) is a progressive and irreversible deterioration of kidney function classified by the last international guidelines into five stages according to glomerular filtration rate [1]. In the last stage (end stage renal disease) the kidney impairment is advanced and cellular/metabolic functions are significantly altered and enable to guarantee normal body homeostasis. Therefore, at this clinical phase, renal replacement therapies (RRTs, peritoneal- or hemo-dialysis) or renal transplantation are needed to ensure patient’s survival.

Although hemodialysis (HD) still represents the leading RRT, in the last years, the number of patients undergoing peritoneal dialysis (PD) procedure is increasing worldwide being a preferable choice for young patients with high life expectancy and an elevated probability to undergo renal transplantation. This is mainly related to a lower activation of microinflammation, compared to HD [2], better preservation of residual renal function [3] and higher quality of life [4].

However, peritoneal catheter and dialysis solutions (characterized by high concentration of glucose, glucose degradation products, low pH and high osmolality) used to remove waste products generated from normal metabolic processes, uremic toxins and to normalize body fluid and electrolytes [5] may still determine the systemic activation of a complex intracellular machinery leading to inflammation and oxidative stress [6]–[8].

In this context, the recruitment, rolling and activation of peripheral blood mononuclear cells (PBMCs) may have a pivotal etiopathogenic role.

As recently reported, transcriptomic analysis of PBMCs of PD patients has shown high expression of important key regulators of inflammation and oxidative stress (e.g. RELA, GSS) in this population [9] with mitochondria having a possible pivotal role in the onset and development of these processes [10], [11].

Mitochondria are essential eukaryotic cells organelles involved in several metabolic pathways, including calcium signaling [12], heme [13] and steroid synthesis [14], apoptosis [15]. This intracellular elements are defined as the “powerhouse of the cell” because of their ability to produce the vast majority of energy necessary for cellular metabolism through the oxidative phosphorylation system (OXPHOS).

Structurally, they present an outer and inner membrane, the latter of which would be impermeable to all molecules in the absence of specific carriers and contains the OXPHOS complexes. The respiratory flux is due to the donation of electrons from NAD- or FAD-dependent substrates, via respiratory chain, to molecular oxygen which is finally reduced to water. Simultaneously, the energy conserving complexes I, III and IV build up a trans-membrane electrochemical gradient by coupling the electron transfer activity to proton translocation from the matrix to the outer side of the inner mitochondrial membrane. Complex V utilizes backward the electrochemical gradient for ATP synthesis. During this process a small percentage of electrons may “leak” from the respiratory chain and partially reduce oxygen, forming superoxide anion (O_2_^−^) [16]. Consequently mitochondria are the major source of ROS in the cell.

Recently increasing evidences showed mitochondrial dysfunction in a broad spectrum of renal disorders [17] and we previously reported, for the first time, that CKD/HD patients exhibited an impaired mitochondrial respiratory system.

Therefore, in order to improve our knowledge about the mitochondria-related cellular changes occurring in the heterogeneous CKD population, to study possible intracellular defense mechanisms against inflammation/oxidative stress and to identify new diagnostic/prognostic biomarkers or therapeutic targets, we decided to analyze in our *in vivo* study some of the key biological cellular regulators of oxidative metabolism in uremic patients in dialysis treatment.

We decided to focus on PD patients since the increasing interest of the nephrological research on this large dialysis patients’ population and because, at present, no study has analyzed the relationship between oxidative stress and mitochondrial deregulation in this population.

## Methods

### Patients and Controls

A total of 30 subjects [n: 15 CKD in peritoneal dialysis (PD) and n:15 healthy subjects (HS)], after signing informed consent, were enrolled in our study. The main demographic and clinical features are summarized in Table 1.

**Table 1:** Demographics and clinical characteristics of study group.

	HS	PD	*p.value*
Number	15	15	*n.s.*
Gender (M/F)	8/7	9/6	*n.s.*
Age (years)	49.9±5.7	51.9±2.5	*n.s.*
Cause of CKD (GN, ADPKD,RVD, ESRD, unknown)	/	6,1,4,1,2	*/*
Time on dialysis (years)	/	2.7±2.1	*/*
BMI (Kg/m^2^)	25±5.0	26.19±3.4	*n.s.*
Systolic blood pressure (mmHg)	120.5±2.7	134.7±7.6	*<0.001*
Diastolic blood pressure (mmHg)	74.2±5.3	80±9.7	*n.s.*
Total protein (g/dl)	7.08±0.3	6.84±0.5	*n.s.*
Albumin (g/dl)	3.9±1.2	3.34±0.6	*n.s.*
Hemoglobin (g/dl)	13.5±1.3	12.27±0.9	*<0.01*
Hs-CRP (ng/ml)	0.69±0.7	12.51±8.5	*<0.001*

Values are expressed as mean ± SD. GN, glomerulonephritis; ADPKD, adult dominant polycystic disease; RVD, renal vascular disease; ESRD, end-stage renal disease. BMI, body mass index; Hs-CRP, high sensitivity- C reactive protein. p-values determined by t-test and Chi-squared test.

Thirteen PD patients have been treated with automated peritoneal dialysis (APD) using a bicarbonate buffer (Physioneal, Baxter) and 2 with a continuous ambulatory peritoneal dialysis (CAPD) using a bicarbonate buffer (Physioneal, Baxter, Chicago, IL, USA).

To avoid confounding factors, all patients suffering from systemic autoimmune disorders, infectious diseases, diabetes, chronic lung diseases, neoplasm, or inflammatory diseases and patients receiving antibiotics, corticosteroids, or non-steroidal anti-inflammatory agents were excluded. No patients had symptomatic coronary artery diseases or a family history of premature cardiovascular diseases.

Serum C-reactive protein (CRP) levels were measured in all patients included in the study using high-sensitivity immunonephelometric (Dade Behring, Marbung, Germany) according to the manufacturer’s protocol.

The study was carried out according to the Declaration of Helsinki and approved by the Institutional Ethic Review Board of the University Hospital “Policlinico di Bari”, Bari, Italy (number of registration: 599/CE).

### Peripheral Blood Mononuclear Cells (PBMCs) Isolation

Fifteen ml whole blood were collected from all subjects included in the study. For PD patients the biological material was obtained during the outpatient clinical evaluations. PBMCs were isolated by density separation over a Ficoll–Paque™ (GE healthcare, Sweden) gradient (460 g for 30 min) and washed three times with PBS pH 7.4/1 mM EDTA (Sigma, Milan, Italy). Cells were counted, and viability was assessed by trypan blue exclusion method (>90% PBMCs were viable).

### RNA Extraction and Real-Time PCR

Total RNA was isolated from PBMCs by RNeasy mini kit Qiagen (QIAGEN AG, Basel, Switzerland). RNA was quantified by Quant-iT RNA Assay kit with Qubit Fluorometer (Invitrogen).

Reverse transcription of RNA was performed using the High Capacity cDNA Reverse Transcription Kit (Applied Biosystems), following the manufacturer’s instructions. One µg of RNA was reverse transcribed using random primer and MultiScribe Reverse Transcriptase. Real-time PCR amplification reactions were performed in duplicate in 20 µl of final volume via SYBR Green chemistry on ABI-Prism 7700 (Applied Biosystem). PCR protocol was performed using QuantiTect Primer Assays (Qiagen, Basel, Switzerland) for PGC-1α, NRF-1, TFAM, MCAD, NRF2, SOD2 and β-actin: 50°C for 2 min, 95°C for 2 min and 40 two-step cycles: 95°C for 15 sec and 60°C for 30 sec. For COX6C, COX7C (subunits of complex IV or Cytochrome c oxidase) and UQCRH (subunit of Complex III or ubiquinol-cytochrome c reductase) the primers were designed by the aid of the Primer3 software (frodo.wi.mit.edu). The forward and reverse primer sequences were: COX6C forward 5′-CTTTGTATAAGTTTCGTGTGG-3′ and reverse 5′-ATTCATGTGTCATAGTTCAGG-3′; COX7C forward 5′-CCCTGGGAAGAATTTGCCA-3′ and reverse 5′-GGAACTGAAACATCCTTATG-3′; UQCRH forward 5′-AGGGACCATTGCGTGGCC-3′ and reverse 5′-AGCTACCAGCCTAAGCCAAA-3′.

Universal master mix obtained from Kapa Biosystems included all reagents. The β-actin gene amplification was used as a reference standard to normalize the target signal. Amplification specificity was controlled by a melting curve analysis and the amount of mRNA target was evaluated using the comparative Ct method.

### Western Blot Analysis for TFAM

Isolated PBMCs from 10 PD patients and 8 HS were lysed in RIPA buffer (1 mM phenylmethylsulphonylfluoride, 5 mM EDTA, 1 mM sodium orthovanadate, 150 mM sodium chloride, 8 µg/ml leupeptin, 1.5% NonidetP-40, 20 mM Tris–HCl, pH 7.4). The lysates were kept on ice for 30 min and centrifuged at 10,000 g at 4°C for 10 min. The supernatants were collected and stored at −80°C until used. Aliquots containing 30 µg of proteins from each lysate were subjected to SDS–PAGE on a Criterion™ *TGX Any kD Stain*-*Free*™ (Biorad) and then electrotransferred onto PVDF membrane (Biorad) by Trans*-*Blot Turbo transfer system (Biorad). The filter was blocked with 5% milk powder in TBS containing 0.1% Tween-20 (TBS-T). Membranes were probed with primary antibody against TFAM (Santa Cruz Biotechnology, Santa Cruz, CA, USA) and incubated with secondary antibody (Santa Cruz Biotechnology, Santa Cruz, CA, USA). Horseradish peroxidase was detected with the ECL-enhanced chemiluminescence system (Amersham, Buckinghamshire, UK). The same membranes were stripped and proteins were rehybridized with anti-β-actin antibody (Santa Cruz Biotechnology, Santa Cruz, CA, USA). Images were acquired using a scanner EPSON Perfection 2580 Photo (EPSON, Long Beach, CA, USA) and quantified by Image J 1.34 Software (http://rsb.info.nih.gov/ij/). The intensity of bands, corresponding to the TFAM protein, was normalized to the β-actin signal.

### Western Blot Analysis for Nuclear Protein Expression of NRF2

Nuclear extracts of freshly isolated PBMCs from 5 randomly selected HS and 5 PD patients were obtained according to the published protocol by Almeida et al [18]. PBMCs were washed with ice-cold PBS and resuspended in buffer A (10 mM HEPES, 10 mM NaCl, 3 mM MgCl_2_, 1 mM EGTA, 0.1% Triton X-100, pH 7.5), supplemented with 50 mM NaF, 1 mM Na_3_VO_4_, 1 mM DTT, 1 mM PMSF, and complete protease inhibitor cocktail (Roche) on ice for 40 min, then centrifuged at 2,400 g for 10 min. The pellets containing nuclei were resuspended in buffer B (25 mM HEPES, 300 mM NaCl, 5 mM MgCl_2_, 1 mM EGTA, 20% glycerol, pH 7.4), supplemented with 50 mM NaF, 1 mM Na_3_VO_4_, 1 mM DTT, 1 mM PMSF, and complete protease inhibitor cocktail on ice for 60 min. The lysates were centrifuged at 12,000 g for 20 min, the supernatants were collected, and the amount of nuclear protein was measured using the Bio-Rad protein assay reagent.

Subsequently, nuclear extracts (20 µg) were resolved in 9% SDS-PAGE and electrotransferred onto nitrocellulose membranes. The filter was blocked with 5% milk powder in TBS-T. Membranes were probed with anti-NRF2 (Genetex GTX103300; 1∶500) and anti-Lamin B (sc-2616, Santa Cruz Biotechnolgy 1∶1000) antibodies. After three washes in TBST, the membranes were incubated with the secondary peroxidase conjugated antibodies. The signal was detected by ECL-enhanced chemiluminescence system (Amersham, Buckinghamshire, UK) according to the manufacturer’s instructions. Images were acquired using a scanner EPSON Perfection 2580 Photo (EPSON, Long Beach, CA, USA) and quantified by Image J 1.34 Software (http://rsb.info.nih.gov/ij/). The intensity of bands, corresponding to the NRF2 protein, was normalized to the Lamin B signal.

### Thiobarbituric Acid Reactive Substances (TBARS) Assay

Plasmatic measurement of Malondialdehyde (MDA) as TBARS was performed for all subjects included in the study using a commercially available kit (AbNOVA Abnova, Walnut, CA) according to the manufacturer’s instructions.

### Resting Metabolic Rate

Resting metabolic rate (RMR) was assessed by indirect calorimetry with the use of an MMC Horizon System 6 (Beckman Sensormedics, Milan, Italy) that measures resting oxygen uptake and resting carbon dioxide production. The subjects were familiarized with the canopy of the calorimeter so that they did not feel suffocated during the measurement period. They were instructed to avoid hyperventilating, fidgeting, and falling asleep. Gas was measured in the morning in the supine position after the subjects had fasted 12 h. Values were considered reliable after a 20-min nonstop period when differences in consecutive values were <5%; at this point, gas measurements were continued for another 20 min, and mean values for resting oxygen uptake and resting carbon dioxide production were calculated. The CV for duplicate measurements in 12 subjects was 5%.

### Statistical Analysis

Results were expressed as mean ± SD. Student’s t-test and Chi-squared test were used to assess differences in clinical, demographic and experimental features. A value of p<0.05 was considered to be statistically significant. R 2.0.1 statistical software was used to perform the above analyses (www.r-project.org).

## Results

### Peroxisome Proliferator-activated Receptor Gamma coactivator 1 Alpha (PGC1-α) and Nuclear Respiratory Factor 1 (NRF-1) Gene Expression

To assess whether PBMCs isolated from CKD patients undergoing PD treatment showed an alteration of the cellular machinery associated to the oxidative energy metabolism, we measured by RT-PCR the expression level of peroxisome proliferator-activated receptor gamma coactivator 1 alpha (PGC1-α) and Nuclear respiratory factor 1 (NRF-1), two genes encoding, respectively, for a transcriptional coactivator and a transcription factor that together stimulate the expression of a broad set of nuclear genes involved in mitochondrial biogenesis and functions.

**Figure 1:**
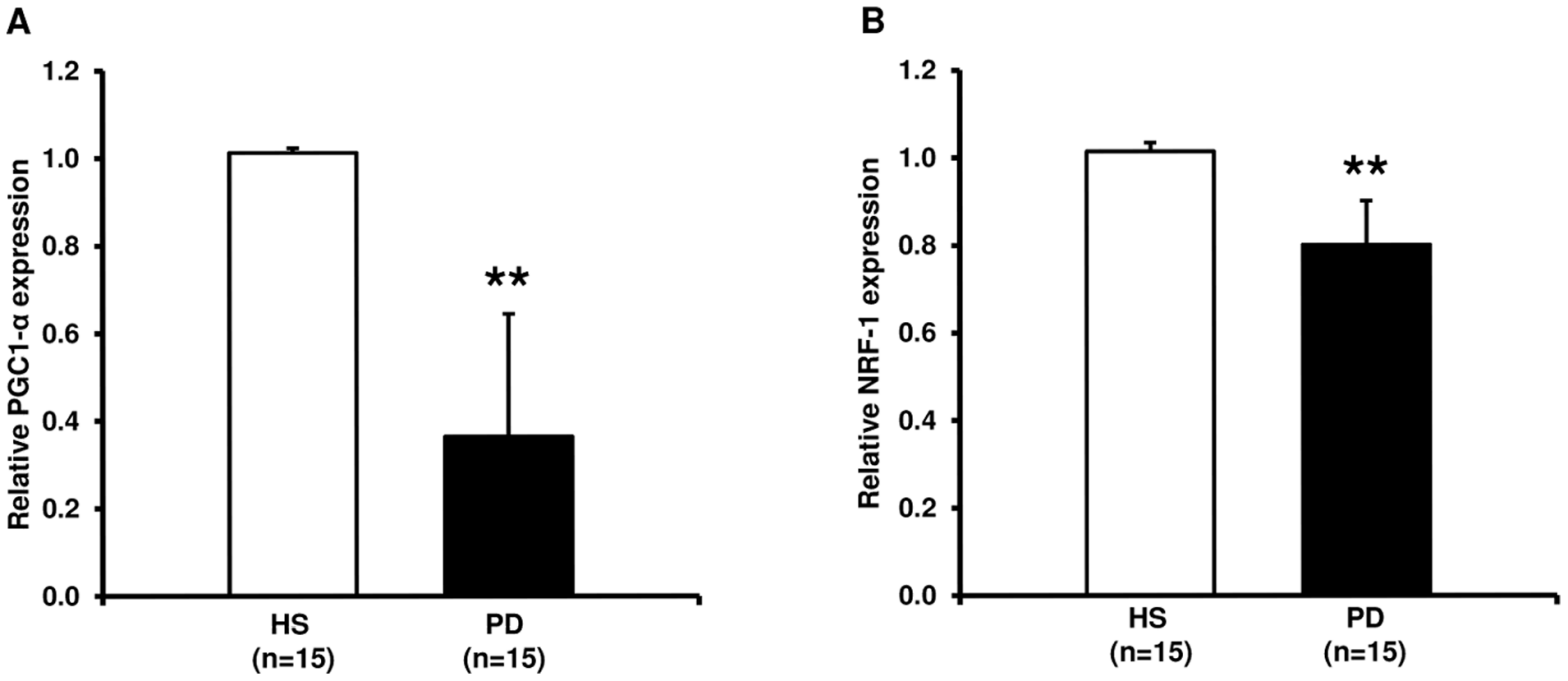
Peroxisome proliferator-activated receptor gamma coactivator 1 alpha (PGC1-α) and nuclear respiratory factor-1 (NRF-1) gene expression by Real-Time PCR in PBMCs from healthy subjects (HS) and chronic kidney disease patients in peritoneal dialysis (PD). The histograms represent the mean ± SD of PGC1-α (A) and NRF-1 (B) expression level, determined by Real-Time PCR in PBMCs from 15 HS and 15 PD patients. For both genes the expression level was significantly lower in PD compared to HS (**p<0.001).

As reported in figure 1, both genes resulted significantly down-regulated in PD compared to HS.

### Mitochondrial Transcription Factor A (TFAM) Gene and Protein Levels

Since TFAM is the major gene regulated by NRF-1 and it regulates mitochondrial transcription and replication, we decided to measure its gene expression in all subjects included in the study.

**Figure 2:**
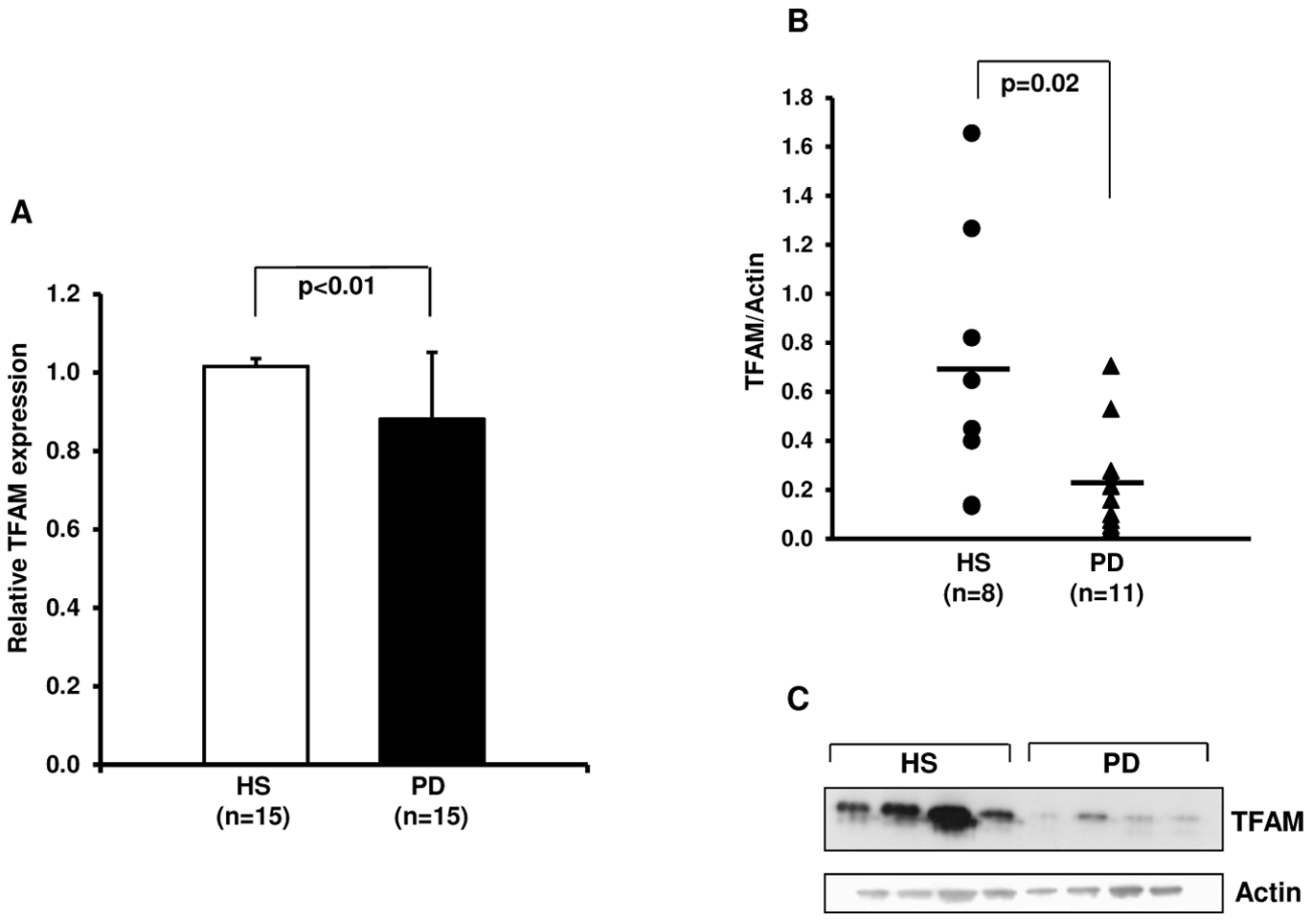
Transcription factor A, mitochondrial (TFAM) gene and protein expression in PBMCs from healthy subjects (HS) and chronic kidney disease patients in peritoneal dialysis (PD). (A) The histogram represents the mean ± SD of TFAM expression level, determined by Real-Time PCR in PBMCs from 15 HS and 15 PD patients. (B) Dot-plot represents the normalized TFAM protein level in total cell lysates of 8 HS and 11 PD patients assessed by Western blotting and (C) the representative western blotting experiment for TFAM. The line represents the mean value. Both TFAM mRNA and protein level was significantly lower in PD patients compared to HS.

As expected, PD patients showed a significant TFAM mRNA down-regulation compared to HS (Figure 2A). Protein analysis by western blot confirmed the results obtained by gene expression (Figure 2B).

### Medium Chain Acyl CoA Dehydrogenase (MCAD) Gene Expression

**Figure 3:**
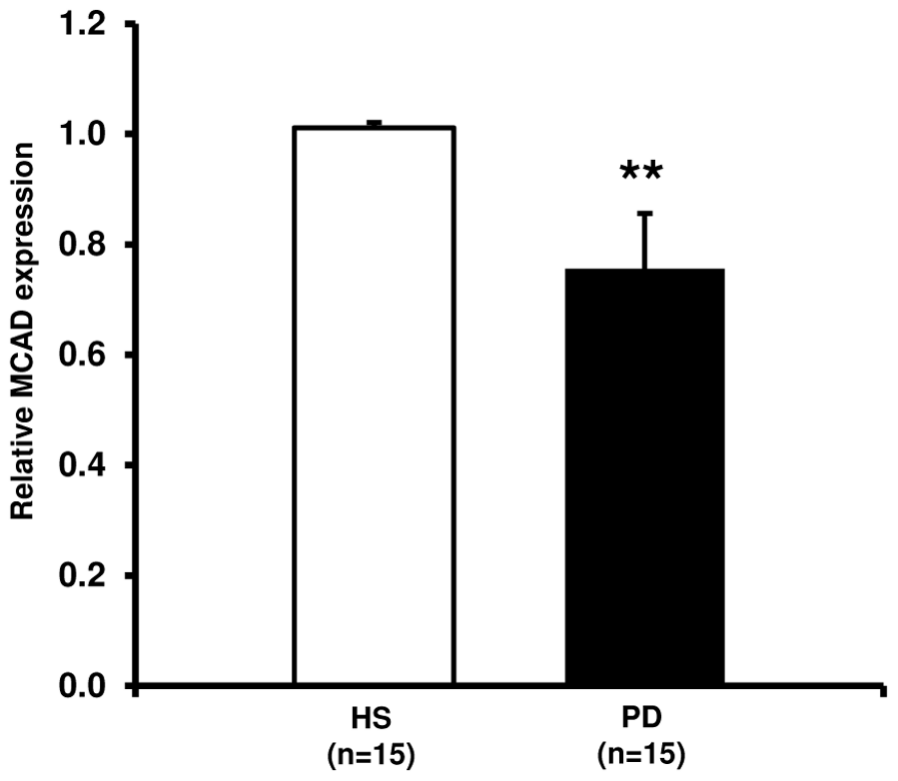
Medium chain acyl CoA dehydrogenase (MCAD) gene expression in PBMCs from healthy subjects (HS) and chronic kidney disease patients in peritoneal dialysis (PD). The histogram represents the mean ± SD of MCAD expression level, determined by Real-Time PCR in PBMCs from 15 HS and 15 PD patients. The expression level was significantly lower in PD compared to HS (**p<0.001).

To confirm the reduced PGC1-α activity we evaluated the mRNA level of MCAD, an oxidoreductase enzyme tightly regulated by this coactivator, that catalyzes the first step of mitochondrial fatty acid beta-oxidation, in our PD patients and HS. MCAD gene expression was significantly lower in PD compared to HS (Figure 3).

### Oxidative Phosphorylation System (OXPHOS) Subunits

**Figure 4:**
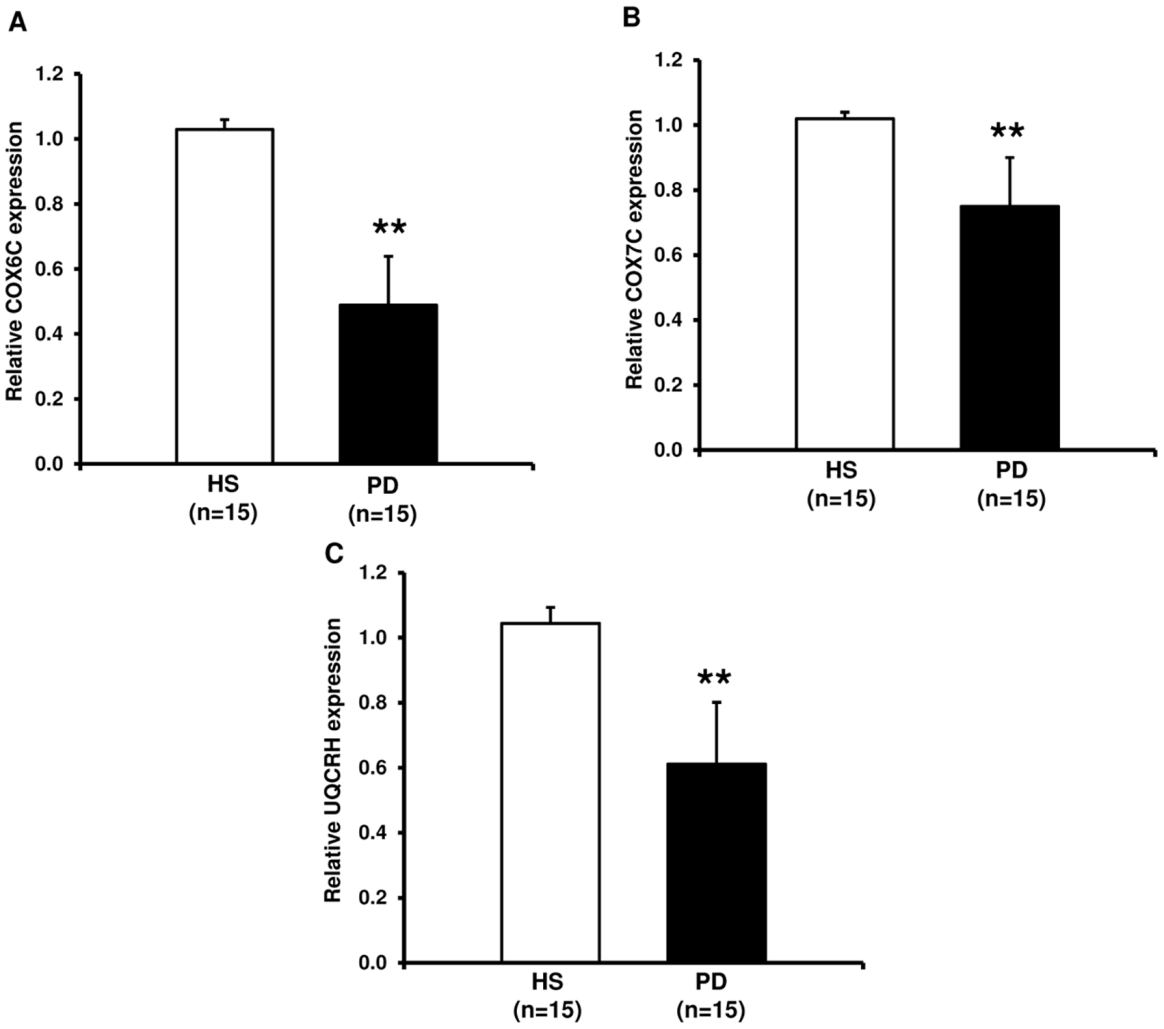
COX6C, COCX7C and UQCRH gene expression by Real-Time PCR in PBMCs from healthy subjects (HS) and chronic kidney disease patients in peritoneal dialysis (PD). The histograms represent the mean ± SD of COX6C (A) COX7C (B) and UQCRH (C) expression level, determined by Real-Time PCR in PBMCs from 15 HS and 15 PD patients. For all these genes the expression level was significantly lower in PD compared to HS (**p<0.001).

Mitochondrial oxidative phosphorylation system (OXPHOS) plays a central role for energy homeostasis in mammals. This machinery is finely regulated by several factors including PGC-1α and NRF-1. Therefore, we decided to analyze whether the down regulation of these factors in our CKD-PD patients induced down-regulation of expression of genes encoding for mitochondrial OXPHOS subunits. Our results revealed that the expression level of genes encoding for UQCRH (complex III subunit) and COX6C/COX7C (complex IV subunits) were significantly hypo-expressed in our PD patients compared to HS (Figure 4). These results suggest a possible reduction of OXPHOS activity.

### Oxidative Stress Evaluation by Thiobarbituric Acid Reactive Substances (TBARS)

**Figure 5:**
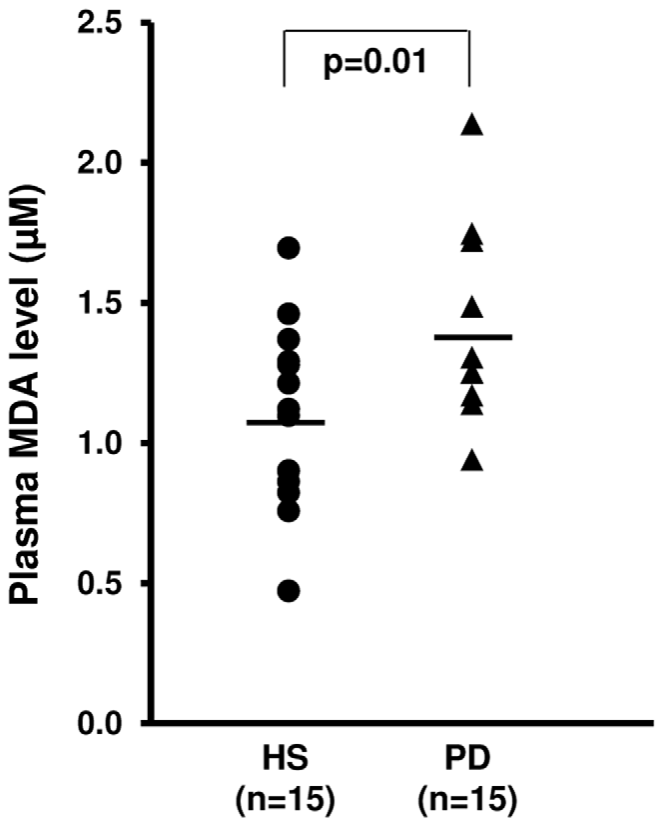
Malondialdehyde (MDA) levels in plasma of healthy subjects (HS) and chronic kidney disease patients in peritoneal dialysis (PD). Dot-plot represents plasma MDA levels in 15 HS and 15 PD patients. Malondialdehyde (MDA) was measured as TBARS. The line represents the mean value. MDA level was higher in PD compared to HS.

To assess the relationship between PCG1-α, NRF-1 deregulation and oxidative stress, we measured the TBARS levels, products of oxidative damage to lipid, in all subjects included in our study. In particular, we analyzed the plasmatic levels of malondialdehyde (MDA), one of several low-molecular-weight end products formed via the decomposition of certain primary and secondary lipid peroxidation products. MDA levels were significantly higher in our PD patients compared to HS (Figure 5) demonstrating an high oxidative stress in our dialysis patients.

### Nutritional Status Evaluation Using the Resting Metabolic Rate (RMR)

**Figure 6:**
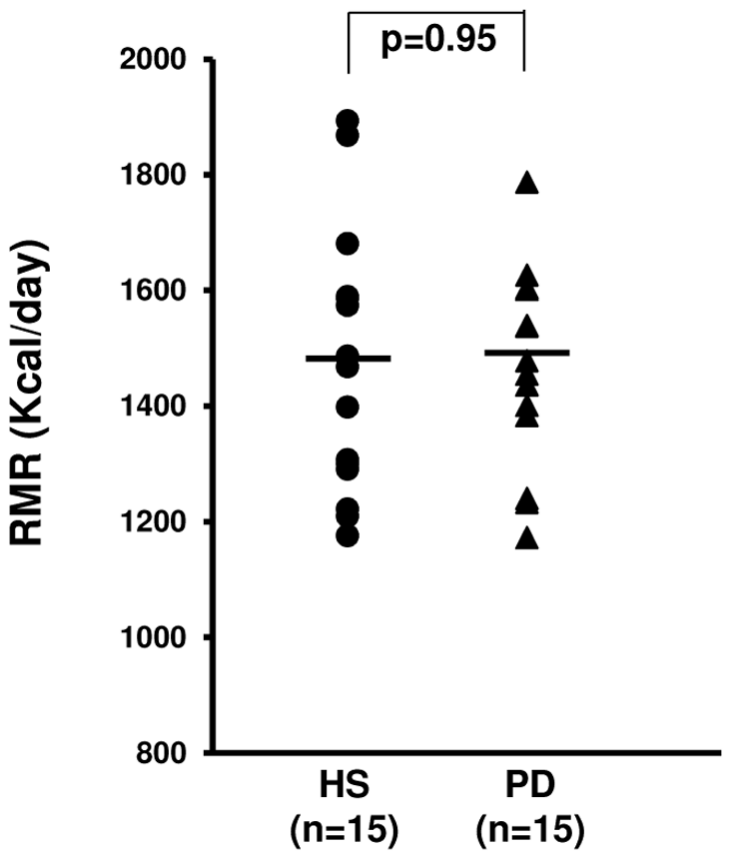
Resting metabolic rate (RMR) assessment in healthy subjects (HS) and chronic kidney disease patients in peritoneal dialysis (PD). Dot-plot represents the RMR assessed by indirect calorimetry in 15 HS and 15 PD patients. RMR levels were similar in the two study groups.

To exclude a possible influence of the nutritional status on the previous results, we performed an indirect calorimetry test to measure the resting metabolic rate (RMR) on all the study population. Concordantly with other reports [19], we did not find any differences between PD and HS (Figure 6).

### Evaluation of the Antioxidant Cellular Response by NRF2 and SOD2 Gene Expression Analysis

Finally, to better define the antioxidant response against the cytotoxic effects of oxidative stress, we evaluated the expression level of Nuclear factor-erythroid-2-related factor 2 (NRF2 or NFE2L2) and Superoxide dismutase 2, mitochondrial (SOD2) in our CKD-PD patients and HS.

**Figure 7:**
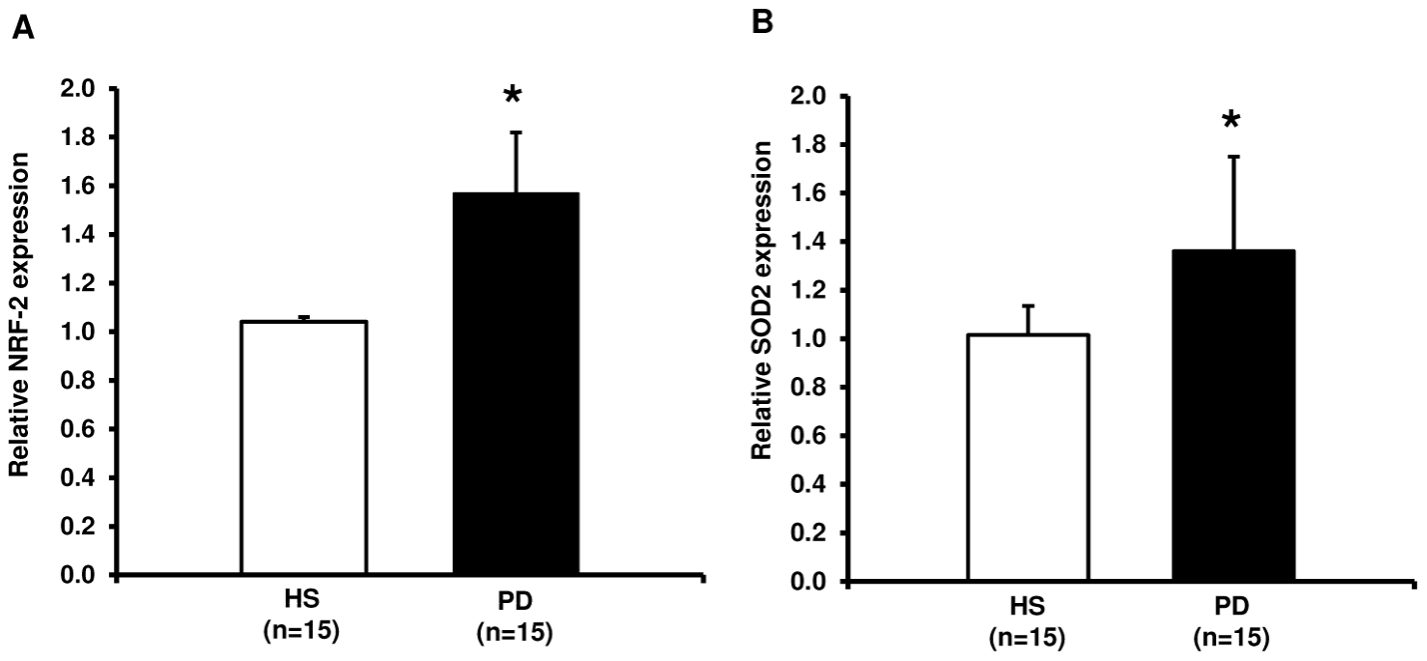
NRF-2 and SOD2 gene expression by Real-Time PCR in PBMCs from healthy subjects (HS) and chronic kidney disease patients in peritoneal dialysis (PD). The histograms represent the mean ± SD of NRF-2 (A) and SOD2 (B) expression level, determined by Real-Time PCR in PBMCs from 15 HS, 15 PD patients. For both genes the expression level was significantly higher in PD compared to HS (*p<0.01).

As predictable, both genes were up-regulated in PD compared to HS demonstrating an enhanced activation of the cellular antioxidant machinery in this study group (Figure 7).

### NRF2 Protein Levels

**Figure 8:**
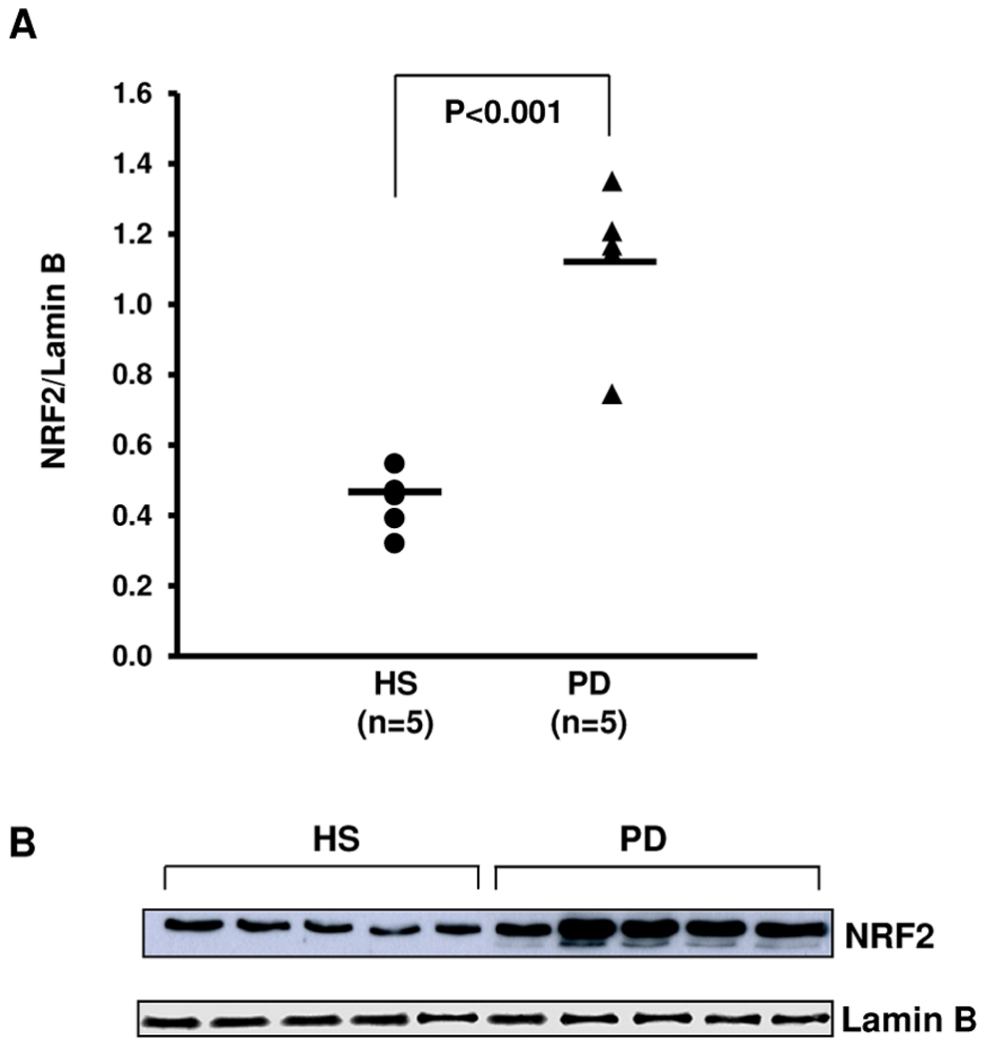
Nuclear factor erythroid-derived 2-like 2 (NRF2) protein expression in nuclear extracts of PBMCs from healthy subjects (HS) and chronic kidney disease patients in peritoneal dialysis (PD). (A) Dot-plot represents the normalized NRF2 protein level in nuclear extracts of 5 HS and 5 PD patients assessed by Western blotting and (B) the representative western blotting experiment for NRF2. The line represents the mean value. NRF2 protein level was significantly higher in PD patients compared to HS.

Because of NRF2 mediates the transcriptional response of cells to oxidative stress by the translocation into nucleus, we evaluated its protein level in nuclear extracts from PBMCs of HS and CKD-PD patients. Concordantly to gene expression analysis, NRF2 protein level resulted significantly higher in CKD-PD patients compared to HS (Figure 8).

## Discussion

Oxidative stress and mitochondrial activity are key elements of many pathological conditions, including neurodegenerative disorders, diabetes, cardiovascular disease and cancer [20]–[23].

Moreover, our research group has recently demonstrated, for the first time, a close link between mitochondrial deregulation and oxidative stress in chronic kidney disease (CKD) patients in conservative and hemodialysis (HD) treatment [24]. In particular, using an innovative high-throughput technology, we discovered that several biological elements involved in the oxidative phosphorylation system and two key constituents of the mitochondrial complex IV (COXI and COXIV) were deregulated in CKD/HD patients compared to healthy controls. In addition, complex IV activity, the terminal enzyme of the mitochondrial respiratory chain catalyzing the electron transfer from reduced cytochrome c to oxygen [25], resulted significantly lower in CKD/HD patients compared to healthy subjects demonstrating a reduced activity of oxidative phosphorylation system in this population. However, at the state of art, the complete cellular mechanism involved in this intricate biological system is still completely unknown.

Therefore, to better comprehend the mitochondria-related biochemical/metabolic cellular alterations in CKD, we decided to use a well standardized biomolecular methodologies (e.g., RT-PCR, western-blotting) to measure the expression level of some key biological cellular regulators of the oxidative metabolism in patients with elevated kidney damage undergoing peritoneal dialysis (PD) treatment.

PD is a renal replacement therapy that, instead of hemodialysis, is associated with better preservation of residual renal function, initial survival advantage, reduced erythropoiesis stimulatory agent requirements and preservation of vascular access sites [26]–[28]. However, the use of “unphysiologic” conventional PD fluids (characterized by acidic pH, high lactate concentrations, high osmolality, high glucose concentrations, and contamination by glucose degradation products) may contribute to the onset/development of several adverse outcomes [29]–[32] and to the activation of oxidative stress [7]. Although well described, the molecular mechanisms associated to latter condition are still not completely known.

To better address this point, we focused on the Peroxisome proliferator-activated receptor gamma coactivator 1 alpha (PGC-1α)-related intracellular machinery. PGC-1α is a well known master regulator of mitochondrial oxidative metabolism [33]. Its expression seems finely tuned to reflect cellular energy needs, with conditions of increased energy demands inducing its expression [34], [35]. PGC-1α performs this task by coactivating a large number of transcription factors, including, among others, nuclear respiratory transcription factor 1 (NRF-1) and in this way, it regulates the activity of numerous nuclear genes encoding mitochondrial proteins [36].

Interestingly, our RT-PCR experiments demonstrated that the expression levels of PGC-1α, NRF-1 and the other analyzed downstream target genes were significantly down-regulated in PD patients compared to HS. Therefore PBMCs of CKD patients showed a specific down-regulation of several nuclear-encoded genes involved in the mitochondrial biogenesis and functions (*TFAM*, *COX6C, COX7C, UQCRH* and *MCAD*).

TFAM has a key biological role because, after migration into mitochondria, it regulates mitochondrial DNA transcription and replication [37]. COX6C and COX7C encode for two subunits of the cytochrome c oxidase (COX or Complex IV), UQCRH is a component of the ubiquinol-cytochrome c reductase complex (complex III), which catalyzes electron transfer from succinate and nicotinamide adenine dinucleotide-linked dehydrogenases to cytochrome c [38]. MCAD, then, is an oxidoreductase enzyme regulated by PGC1-α, that catalyzes the first step of mitochondrial fatty acid beta-oxidation [39].

Based on previous literature evidences reporting that reactive oxygen species may induce PGC-1α down-regulation [40], we assumed that this biological/biochemical complex, through a mitigation of the mitochondrial OXPHOS activity, could represent a protective adaptive response against chronic cellular perturbation associated to the kidney disease-related oxidative injury.

Our CKD patients, in fact, showed higher plasma concentration of Malondialdehyde (MDA), a thiobarbituric acid reactive substance (TBARS) commonly known as a marker of oxidative stress [41], compared to healthy controls.

Additionally, our results confirmed previous literature evidences reporting that advanced CKD “*per se*” mainly through the accumulation of several circulating uremic toxins (e.g., indoxyl sulfate, p-cresyl sulfate) [42], [43] and the interaction of PBMCs with bio-incompatible dialysis devices can cause their activation with imbalance between pro- and anti-oxidant activities resulting in high oxidative stress [8], [44].

This hypothesis was also in part confirmed by our finding of an additional activated cellular anti-oxidant machinery in PBMCs of PD patients. Specifically, Nuclear factor erythroid-derived 2-like 2 (NRF2 or NFE2L2), a transcription factor regulating the expression of numerous antioxidant/detoxifying enzymes, and one of its down-stream target genes superoxide dismutase-2 mitochondrial (SOD2) [45] resulted significantly up-regulated in our CKD-PD population. SOD2 binds to the superoxide byproducts of oxidative phosphorylation and converts them to hydrogen peroxide and diatomic oxygen [46].

**Figure 9:**
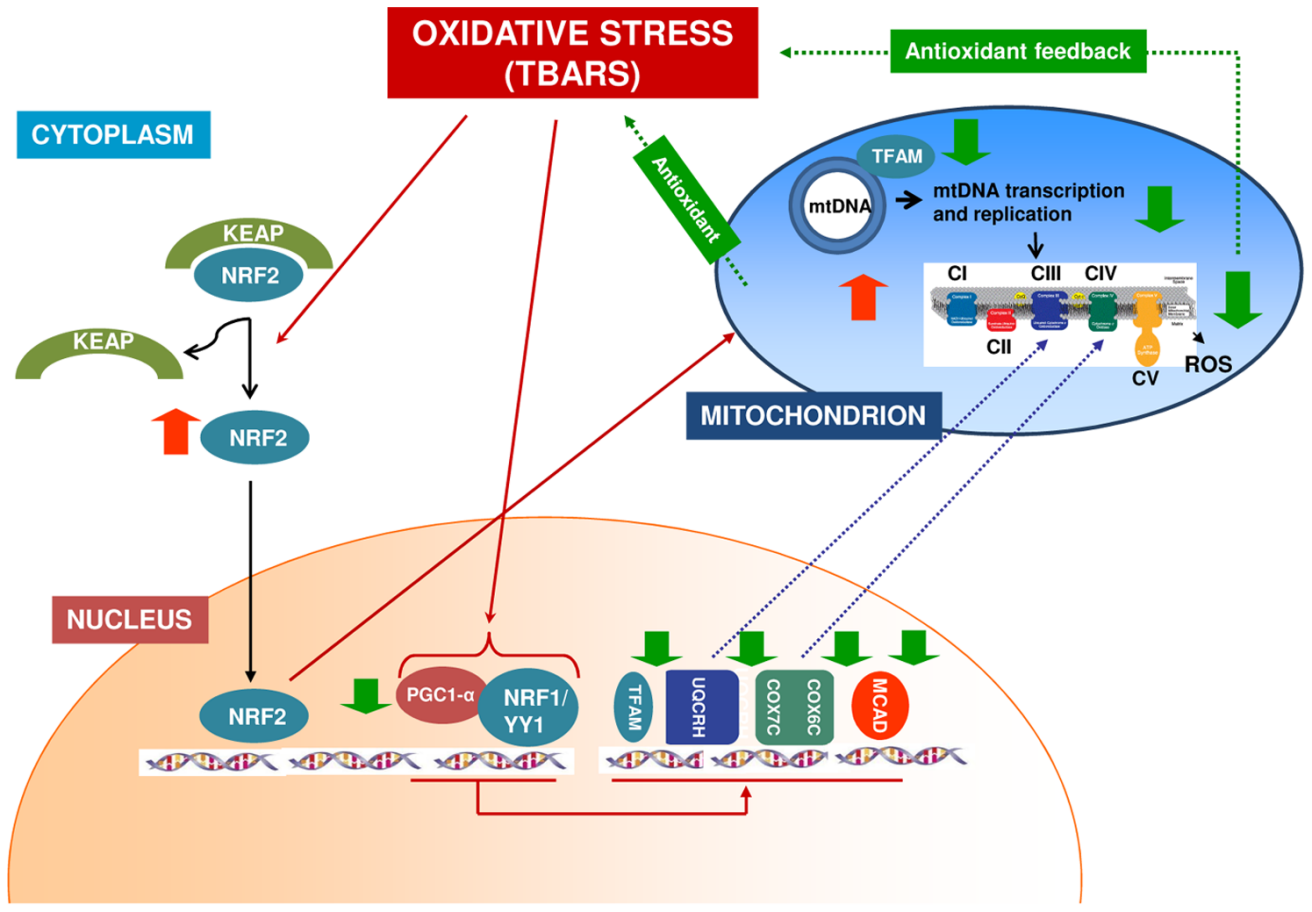
Supposed mechanism of PGC1-α/NRF1-NRF2 pathway anti-oxidative stress cellular defense in chronic kidney disease patients in peritoneal dialysis treatment. Oxidative stress alters the interaction of Kelch-like ECH-associated protein 1 (Keap1) and Nuclear factor erythroid-derived 2-like 2 (Nrf2), thereby liberating Nrf2 activity from repression by Keap1. NRF2 migrates into the nucleus were it activates the transcription of Superoxide dismutase 2, mitochondrial (SOD2). At the same time, oxidative stress causes the down-regulation of Peroxisome proliferator-activated receptor gamma coactivator 1 alpha (PGC1-α) and Nuclear respiratory factor-1 (NRF-1) with the consequent down-regulation of PGC-1α downstream target genes (TFAM, COX6C, COX7C, UQCRH and MCAD). The reduced TFAM expression causes a decrease in mitochondrial transcription and replication. The down-regulation of all these factors suggests the decrease in mitochondrial OXPHOS activity in order to reduce ROS accumulation and creating an antioxidant feedback.

Therefore, all together, our results, although generated on a small but well selected patients’ population, revealed, for the first time, a fine regulated intracellular biochemical system associated to oxidative stress response in CKD patients. It is plausible that this redox-dependent mechanism could have a pivotal role in antioxidant defense cellular strategy occurring in cells of these patients (Figure 9). However, further studies are necessary to better delineate all the biological/biochemical mechanisms involved.

The main limitation of our study is the lack of the analysis of all potential clinical variables able to influence the “mitochondrial” transcriptomic profile primarily due to the time and cost consuming of a global analytic/research strategy.

Moreover, our in vitro model failed to corroborate our in vivo findings (See File S1 and Figure S1, 2, 3 and 4). In fact, PBMCs stimulated with high glucose PD dialysis solutions showed the up-regulation of all the previous analyzed genes. These contradictory results clearly demonstrated the unquestionable complexity of this machinery in which all together uremia, microinflammation, subclinical/clinical peritoneal infections, acidosis, electrolytic unbalance contribute to the onset and development of this biological/clinical condition. Therefore, we strong encourage a collaborative international research program to address these points.

Finally, we can not exclude that, in future, the modulation of this machinery could turn on as a valuable point of therapeutic intervention to reduce oxidative stress-related clinical complications in CKD patients in both conservative and dialysis treatment.

## Supporting Information

Figure S1 **Peroxisome proliferator-activated receptor gamma coactivator 1 alpha (PGC1-α), nuclear respiratory factor-1 (NRF-1) and Transcription factor A, mitochondrial (TFAM) gene expression in PBMC from 3 healthy subjects stimulated with PD fluid.** The histograms represent the mean ± SD of PGC1-α (A), NRF-1 (B) and TFAM (C) level of expression, determined by Real-Time PCR in PBMC from 3 HS stimulated with PD fluid. All the three genes resulted significantly up-regulated after 6 and 24 h of incubation with PD fluid (#p<0.05; ##p<0.01 versus CTR).
(TIF)

Figure S2 **Medium chain acyl CoA dehydrogenase (MCAD) gene expression in PBMC from 3 healthy subjects stimulated with PD fluid.** The histogram represents the mean ± SD of MCAD level of expression, determined by Real-Time PCR in PBMC from 3 HS stimulated with PD fluid. The expression level was significantly up-regulated after 6 and 24 h of incubation with PD fluid (#p<0.05 versus CTR).
(TIF)

Figure S3 **COX6C, COCX7C and UQCRH gene expression in PBMC** **from 3 healthy subjects stimulated with PD fluid.** The histograms represent the mean ± SD of COX6C (A) COX7C (B) and UQCRH (C) level of expression, determined by Real-Time PCR in PBMC from 3 HS stimulated with PD fluid. All the three genes resulted significantly up-regulated after 6 and 24 h of incubation with PD fluid (#p<0.05; ##p<0.01 versus CTR).
(TIF)

Figure S4 **NRF-2 and SOD2 gene expression by Real-Time PCR in PBMC from 3 healthy subjects stimulated with PD fluid.** The histograms represent the mean ± SD of NRF-2 (A) and SOD2 (B) level of expression, determined by Real-Time PCR in PBMC from 3 HS stimulated with PD fluid. Both genes were up-regulated after 6 and 24 h of incubation with PD fluid (#p<0.05; ##p<0.01 versus CTR).
(TIF)

File S1 Supplementary methods.
(DOC)
