# Supplementary material for: Downregulation of Nuclear-Encoded Genes of Oxidative Metabolism in Dialyzed Chronic Kidney Disease Patients
Source: PLoS One. 2013 Oct 28;8(10):e77847. doi: 10.1371/journal.pone.0077847 (PMC3810143; doi:10.1371/journal.pone.0077847)
Supplement: File S1 — Supplementary methods. (DOC) [file pone.0077847.s005.doc]

**Supplementary methods**

*Stimulation of PBMC with PD fluid*

PBMCs, isolated from 3 healthy subjects (HS), were resuspended in RPMI-1640 (Sigma) supplemented with 2 mM L-glutammine (1%), penicillin (100 U/ml), and streptomycin (100 μg/ml), and stimulated with peritoneal dialysis (PD) fluid (3.86% glucose, Physioneal, Baxter) for 6 and 24 h.

After extraction, 1 µg of RNA was reverse transcribed into cDNA and real-time PCR was performed (as previously described) to measure the expression level of the following genes: *PGC-1α, NRF-1, TFAM, MCAD, NRF2, SOD2, COX6C, COX7C* and *UQCRH* before (CTR) and after PD fluid treatment.
